# Severe Perinatal Presentations of Günther’s Disease: Series of 20 Cases and Perspectives

**DOI:** 10.3390/life14010130

**Published:** 2024-01-17

**Authors:** Claire Goudet, Cécile Ged, Audrey Petit, Chloe Desage, Perrine Mahe, Aicha Salhi, Ines Harzallah, Jean-Marc Blouin, Patrick Mercie, Caroline Schmitt, Antoine Poli, Laurent Gouya, Vincent Barlogis, Emmanuel Richard

**Affiliations:** 1 Pediatric Haematology Department, Timone Enfant, Assistance Publique Hôpitaux de Marseille (APHM), F-13005 Marseille, France; claire.goudet@ap-hm.fr (C.G.); audrey.petit@ap-hm.fr (A.P.); vincent.barlogis@ap-hm.fr (V.B.); 2 Department of Biochemistry, Groupe Hospitalier Pellegrin, CHU Bordeaux, F-33076 Bordeaux, France; cecile.ged@chu-guadeloupe.fr (C.G.);; 3 BRIC, Bordeaux Institute of Oncology, Inserm UMR1312, University of Bordeaux, 146 Rue Léo Saignat, F-33076 Bordeaux, France; 4 Laboratory of Excellence Gr-Ex, Institut Imagine, F-75015 Paris, Franceantoine.poli@aphp.fr (A.P.); laurent.gouya@inserm.fr (L.G.); 5 Neonatology and Pediatric Haematology, CHU de Montpellier, F-34295 Montpellier, Francep-mahe@chu-montpellier.fr (P.M.); 6 Faculté de Médecine d’Alger, Department of Dermatology, 16010 Alger, Algeria; salhi2002dz@yahoo.fr; 7 Genetic Department, CHU de Saint-Etienne, F-42055 Saint-Etienne, France; ines.harzallah@chu-st-etienne.fr; 8 Centre de Compétence Maladies Rares Porphyries, Groupe Hospitalier Saint-André, CHU Bordeaux, F-33000 Bordeaux, France; 9 Department of Internal Medicine and Clinical Immunology, Groupe Hospitalier Saint-André, CHU Bordeaux, F-33000 Bordeaux, France; 10 Centre de Recherche sur l’Inflammation, Université de Paris Cité, Inserm U1149, F-45018 Paris, France; 11 Assistance Publique-Hôpitaux de Paris, Centre de Référence Maladies Rares Porphyries, Hôpital Louis Mourier, F-92400 Colombes, France

**Keywords:** erythropoietic porphyria, congenital erythropoietic porphyria, UROS deficiency, Günther’s disease, hemolytic anemia, hydrops fetalis, malformative syndrome, phenotypic variability

## Abstract

(1) Background: Congenital erythropoietic porphyria (CEP), named Günther’s disease, is a rare recessive type of porphyria, resulting from deficient uroporphyrinogen III synthase (UROS), the fourth enzyme of heme biosynthesis. The phenotype ranges from extremely severe perinatal onset, with life-threatening hemolytic anaemia, to mild or moderate cutaneous involvement in late-onset forms. This work reviewed the perinatal CEP cases recorded in France in order to analyse their various presentations and evolution. (2) Methods: Clinical and biological data were retrospectively collected through medical and published records. (3) Results: Twenty CEP cases, who presented with severe manifestations during perinatal period, were classified according to the main course of the disease: antenatal features, acute neonatal distress and postnatal diagnosis. Antenatal symptoms (seven patients) were mainly hydrops fetalis, hepatosplenomegaly, anemia, and malformations. Six of them died prematurely. Five babies showed acute neonatal distress, associated with severe anemia, thrombocytopenia, hepatosplenomegaly, liver dysfunction, and marked photosensitivity leading to diagnosis. The only two neonates who survived underwent hematopoietic stem cell transplantation (HSCT). Common features in post-natal diagnosis (eight patients) included hemolytic anemia, splenomegaly, skin sensitivity, and discoloured teeth and urine. All patients underwent HSCT, with success for six of them, but with fatal complications in two patients. The frequency of the missense variant named C73R is striking in antenatal and neonatal presentations, with 9/12 and 7/8 independent alleles, respectively. (4) Conclusions: The most recent cases in this series are remarkable, as they had a less fatal outcome than expected. Regular transfusions from the intrauterine period and early access to HSCT are the main objectives.

## 1. Introduction

Congenital erythropoietic porphyria (CEP; OMIM 263700), also named Günther’s disease, is a rare inborn error of heme biosynthesis, due to uroporphyrinogen III synthase (UROS; EC 4.2.1.75) deficiency [1]. The disease is caused by the overproduction of non-physiologic type I porphyrin isomers by the erythron and is characterized by skin photosensitivity and variable degrees of chronic hemolytic anemia. The uroporphyrin and coproporphyrin isomers type I are photo-activated and undergo auto-oxidation to the corresponding porphyrins, causing erythrocyte damage and tissue deposits. Upon exposure to sunlight and other sources of long-wave ultraviolet light, the photocatalytic compounds elicit a phototoxic reaction, resulting in blistering and vesicle formation with the increased fragility of the skin [2,3].

In most cases, CEP is caused by *UROS* gene pathogenic variants and follows an autosomal recessive inheritance pattern. One specific variant of the GATA1 gene, located on the X chromosome, has also been linked to the CEP phenotype in male patients [4].

Clinical manifestations are markedly heterogeneous, ranging from non-immune hydrops fetalis to milder, later-onset forms characterized by moderate cutaneous involvement, without haematologic symptoms in adult life. Severely affected patients are transfusion-dependent with secondary hypersplenism; severe cutaneous lesions are worsened by chronic infection and bone resorption, leading to loss of digits and dramatic facial deformities [1,3]. Disease severity is modulated by the level of porphyrin excess and known to be influenced by the interaction with the previous steps of heme biosynthesis, i.e., ALAS2 (EC 2.3.1.37), the limiting enzyme of the pathway and the target of iron-dependent regulatory proteins [5].

The management of severe CEP patients had been limited to supportive measures, including the strict avoidance of sun and light exposure, sunscreen lotions, transfusion program, and correction of bone demineralisation, ocular complications and scared areas of face and skin, until the first successful hematopoietic stem cell transplantation [6]. Novel therapeutic options have been investigated in animal models and/or moderate forms without hemolysis, through the modulation of protein folding and stability or by targeting the iron supply via substrate reduction therapy [7,8,9,10].

Here, we present the national cohort of all perinatal cases of CEP with a genetic diagnosis in France in order to analyse its various presentations and evolution.

## 2. Materials and Methods

This study included all perinatal CEP cases recorded by the French Center for Porphyria whose diagnosis was confirmed by genetic analysis between 1990 and 2021. Clinical and biological data were retrospectively collected through medical records and published reports. Previously published cases and the corresponding references are indicated in Table 1, Table 2 and Table 3. *UROS* gene analysis was mainly performed by Sanger sequencing at the Biochemistry Department of Bordeaux University Hospital, or recently through the use of large-scale simultaneous sequencing using national whole-exome sequencing (WES) platforms. Informed consent was obtained for the genetic analyses of each case mentioned in the study.

## 3. Results

Twenty CEP cases belonging to seventeen families (families VII, XIII and XIV; 13 included two different cases, named case 1 and 2) presenting with severe manifestations in the perinatal period were gathered and classified according to the main course of the disease in three situations: antenatal features, acute neonatal distress, and post-natal diagnosis [6,11,12,13,14,15,16,17,18,19,20,21,22,23].

**Table 3:** Main characteristics of severe post-natal forms of CEP.

Post-Natal Cases	Erythrodontia	Photosensitivity	Hirsutism	Splenomegaly	Hepatomegaly	Hemolytic Anemia	Thrombopenia	Liver Dysfunction	Porphyrins Analysis	UROS Genotype	Age at Diagnosis (Weeks of Gestation)	Age at HSCT	Issue	References
Family/ (Cases)	Main Pathological Features					Biological Findings			Diagnosis		Outcome			
I	yes	yes	yes	yes	yes	yes			Bd, Ur	T228M/T228M	NA	4 y	success	[18]
III	yes	yes		yes	yes	yes		yes	Bd, Ur, Enz	C73R/Q187P	8 Mo	22–30 Mo	success	[6,21,22]
VI	yes	yes		yes	yes	yes	yes	yes	Bd, Ur	C73R/C73R	8 Mo	22–24 Mo	success	[6,20,23]
VII (1 and 2)	yes	yes		yes		yes			Bd, Ur	A69T/A69T	12–18 Mo	4 y	success	[19]
IX	yes	yes		yes	yes	yes	yes	yes	Bd, Ur, Enz	A69T/C73R	6 Mo	10 Mo	success	[6]
X	yes	yes		yes		yes	yes	yes	Bd, Ur, Enz	L4F/G225S	6 Mo	11 Mo	fatal	[6]
XII	yes	yes		yes		yes		yes	Bd, Ur, Enz	A69T/V82F	8 Mo	16 Mo	fatal	[6]

*UROS* genotype cf Table 4; Bd, blood; Enz, erythrocyte enzyme assay; Ur, urine; Mo, months; y, years.

### 3.1. Antenatal Presentation

Antenatal presentation concerned seven patients from six pedigrees (Table 1). Hydrops fetalis was noticed from 17 to 20 weeks of gestation (WG) in the first cases, and even earlier (12 weeks) in the most recent case with fetal ascites (personal communication). Anemia and hepatosplenomegaly were commonly associated. Abortion was the main outcome, either spontaneous or medical. The first described case (II) received two transfusions in utero before a premature birth. A unique baby (XVI) was also rescued from premature birth at 35 WG (personal communication).

A retrospective analysis of foetopathological data revealed the frequency of mild to severe malformations, associated with diffuse edemas and growth retardation [14]. Even if they are difficult to characterize, the mild dysmorphic features that could be observed in three cases (IV, VIII, XVII) are as follows: ocular hypertelorism, up-slanting palpebral fissures, short nose and anteverted nostrils, low-set ears, micrognathia, macroglossia, or micromelia of the upper limb. Two fetuses had a severe polymalformative syndrome: abnormal bowel rotation, Arantius canal agenesis, renal hypotrophy, pulmonary sequestration (XIV-2), macrosomia and ascites, left superior vena cava, corpus callosum agenesis, plagiocephaly, sexual ambiguity including hypospadias, and cryptorchidia (XVI, the only viable case of the antenatal group).

The diagnosis of CEP was mainly retrospective, relying mostly on *UROS* gene analysis for recent cases. A common missense variant of *UROS* gene (p.C73R) was present in all cases, and homozygous in half of them.

### 3.2. Acute Neonatal Presentation

Acute neonatal distress was at the foreground in five near-full-term babies from four distinct families, as depicted in Table 2 [6,15,16,17]. Non-specific multi-organ decompensation, characterized by respiratory distress, hemodynamic shock, hemorrhage, hemolytic anemia, and liver dysfunction, is common to all cases. The diagnosis was guided by the occurrence of skin lesions on UV-exposed areas due to severe photosensitivity and confirmed by porphyrin analysis in blood and urine. An unfavorable trend occurred in three patients before the end of the first trimester of life due to multi-organ failure. Antenatal manifestations could have been underestimated in these cases, due to a less accurate pregnancy monitoring.

Little information could be retrieved about the antenatal period in patient V. A prenatal diagnosis based on amniotic fluid was performed for the next pregnancy and confirmed the diagnosis of CEP, which resulted in medical abortion. In patient XI, antenatal ultrasounds were considered normal, but respiratory distress occurred at delivery. Anemia, thrombocytopenia, and hepatosplenomegaly with liver failure were accompanied by severe photosensitivity upon phototherapy, and purple-colored urine quickly led to CEP diagnosis.

The third-trimester ultrasonography detected growth retardation in siblings XIII, with hydrops fetalis and partial corpus callosum agenesis, in patient XIII-2. Cytomegalovirus infection was the first provisional diagnosis in patient XIII-1, where the occurrence of cutaneous lesions, at day 10, evoked bullous epidermolysis, until porphyrin analysis, and *UROS* genotyping, performed in the next affected newborn (XIII-2), changed the diagnosis.

The most recent case (XV) was a challenging diagnosis. A malformative anomaly was evoked upon antenatal routine ultrasonography, showing increased nuchal translucency and right ventricular hypertrophy. Full-term delivery was achieved via c-section, due to abnormal fetal heart tracing. Initial respiratory distress, hepatosplenomegaly, ecchymosis and transient red urine were associated with hemolytic anemia and thrombocytopenia. Bone marrow aspirate was performed upon the initial suspicion of familial hemophagocytic lymphohistiocytosis. Expert scrutiny allowed for the identification of porphyrin crystals in the hyperplasic erythroid lineage, followed by porphyrin analysis in blood and urine. In the meantime, WES identified homozygous *UROS* gene variant (C73R), which was shared by three of the four families with severe neonatal distress. Only two neonates rescued from initial decompensation were maintained by regular transfusion regimen, before curative hematopoietic stem cell transplantation (HSCT) at the age of 10 months. Graft rejection necessitated a second HSCT in patient XI. Follow-up was more recent in patient XV.

### 3.3. Late Post-Natal Presentation

Post-natal diagnosis concerned eight cases from seven families (Table 3). All patients were previously published and underwent HSCT [6,18,19,20,21,22,23]. Common features include hemolytic anemia, splenomegaly, skin photosensitivity, and discolored teeth and urine due to porphyrin accumulation. Liver dysfunction (cytolysis, cholestasis) was described in most patients (6/8). Age at diagnosis ranged from 6 to 18 months, depending on the duration of sun exposure. Porphyrin analyses in blood urine and feces, together with UROS enzymatic assay, were the main diagnostic argument, which was further confirmed by *UROS* gene sequencing.

Chronic hemolysis was the main criteria for HSCT, and the only curative measure for multi-organ involvement due to the accumulation of photo-activated porphyrins. HSCT was performed as early as 10 months, as soon as a compatible donor was available, mainly an unrelated donor, in the first patients. Significant morbi-mortality was observed, with a fatal issue caused by delayed liver failure in two patients despite full correction of the phenotype in all patients. Three different French centers were involved in this treatment: in Strasbourg (the first French description, family I, [18], Lyon (two children from the same family, VII, [19], and Paris for the remaining six recently reviewed patients [6].

A close analysis of the published reports concerning the patients classified as ‘post-natal diagnosis’ revealed uncommon manifestations in the antenatal and/or neonatal period in family III and VI. Hepatosplenomegaly and transient liver dysfunction, including cholestasis and cytolysis, was noted 30 h post delivery in patient III, while the first dermatological advice for skin photosensitivity and nail dysplasia took place at 7 months [21,22]. Mild fetal ascites was described during pregnancy for patient VI, at 27 WG, which was explored by amniotic puncture at weeks 28, 30, and 37, showing mild anemia (9.5–10.1 g/dL) and thrombopenia (28–29 Giga/L). A mild decrease in ascites volume occurred during follow-up. Delivery was performed at 37 WG, via c-section, of a eutrophic newborn with hepatomegaly, splenomegaly, and mild ascites, with no pleuro-pericardial effusion or oedema. Thrombopenia and transient liver dysfunction were monitored until day 10. Normalisation of biological parameters was noticed during the first trimester of life, except for mild anemia (11 g/L). Skin eruption and bullous lesions causing scarring upon solar exposure occurred from the age of three months [23].

Of note, the two patients (III, VI) shared the common severe allele ‘C73R’, either homozygous (III), or compound heterozygous (VI), as opposed to the various alleles presented by the remaining patients in the series (Table 4).

The main data concerning porphyrin accumulation at diagnosis in the three patient classes are reported in Table 5. Whatever the biologic samples tested, (blood, urine, feces, amniotic or ascites fluid), unequivocal results were obtained with the massive accumulation of porphyrins, identified as mainly being coproporphyrin and uroporphyrin type I isomers. When amenable, residual UROS enzymatic activity was barely detectable or profoundly reduced (from <1 to 20% to the normal rate). Due to missing data and the dispersed technical means used in the whole series, the correlation between porphyrin accumulation, residual UROS activity, and genotype could not be drawn.

*UROS* gene analysis (Table 1, Table 2, Table 3 and Table 4) showed a striking frequency of the missense variant named C73R in antenatal and neonatal presentations, 9/12 and 7/8 independent alleles, respectively, compared to the postnatal forms (4/14). Of note, homozygosity for C73R variant occurred independently of consanguineous union (present family XIII only). All parents were of Caucasian origin, which is associated with a high prevalence of this severe variant. The missense c.205G > A; p.(Ala69Thr) was shared by three unrelated families (VII, IX, XII), showing unusual frequency (3/14). Other variants included various missense (including a position not yet reported: c.224A > G, XVII), an exonic substitution causing a splicing defect (c.244G > T), and a substitution located on a critical zone of the *UROS* gene promoter (c.-70C > T) [24]. Due to sample exhaustion, the second deleterious allele could not be determined in family IV.

## 4. Discussion

Congenital erythropoietic porphyria, or Günther’s disease, is one of the rarest porphyria, and affects all ethnic groups. The wide variability in the age of onset and clinical severity may contribute to the delayed diagnosis, although, in most cases, the severe photosensitivity developed soon after birth is suggestive of this. The pink or red-brown staining of diapers due to large amounts of urinary porphyrins may be the first clue regarding the disease in infancy. Additionally, erythrodontia, a pinkish-brown discoloration of the teeth due to porphyrin deposition in the dentin, may attract attention. The urinary excretion of heme precursors (aminolevulinate, ALA, and porphobilinogen (PBG)) is usually within the normal range. Increased concentrations of uroporphyrin and coproporphyrin type I isomers in erythrocytes, urine, and feces are the biochemical hallmark of the disease.

The major debilitating clinical features are cutaneous photosensitivity and anemia. Exposure to sunlight causes a vesicular eruption that can lead to erosions, infections, and scarring. Repetitive injury may lead to mutilation, especially of the ears, nose, and digits. Hypertrichosis is commonly observed on the upper arms and the face. Anemia due to hemolysis can be severe, and some individuals are transfusion-dependent. Splenomegaly secondary to hemolysis is common, and iron overload can impair liver function.

A number of factors can lead to the phenotypic variability in CEP, including the level of residual UROS activity, the degree of hemolysis, the extent of reactive erythropoiesis, and exposure to ultraviolet light [1,2,3].

Little information has been gained concerning the physiopathology of severe forms with perinatal manifestations, partly due to the challenges regarding accurate diagnosis in life-threatening situations. Mouse models of the disease harboring the common UROS variant ‘C73R’ had poor viability, hampering decisive experiments, while less severe forms were mainly used for gene therapy purposes [25]. An analysis of the available foetopathology records shows the frequency of developmental defects reminiscent of those observed in the dyserythropoietic conditions encountered in congenital anemias.

Severe tissue hypoxia caused by anemia is the leading factor involved in the malformative syndrome observed in 2/3 of the neonates diagnosed with alpha-thalassaemia. [26].

Stress erythropoiesis is a prominent feature in severe CEP, resulting from the combination of a reduced survival time for erythrocytes and ineffective erythropoiesis [2]. Studies performed in a mouse model of CEP have analyzed the respective contribution of bone marrow and spleen erythropoiesis in compensating for hemolytic anemia [27]. In the bone marrow, the results suggested an increased maturation rate and cellular exit (homeostatic erythropoiesis). Stress erythropoiesis resulted in enlarged spleens containing large numbers of erythroblasts at all stages of maturation, but decreased apoptosis, as opposed to the ineffective erythropoiesis observed in chronic hemolytic anemias such as thalassemias. In humans, a histological exam of fetal tissue may detect hyperactive extramedullary erythropoiesis in spleen and liver, while bone demineralization is secondary to bone marrow hyperplasia. Iron overload, a consequence of chronic hemolysis and the impaired iron balance caused by anemia, may contribute to liver dysfunction and multi-organ failure.

The biochemical diagnosis of CEP is usually established by the detection of markedly elevated levels of uroporphyrin I and coproporphyrin I in any available biological sample, mostly urine and erythrocytes in living neonates, and eventually amniotic or ascites fluid. Of note, illumination under Wood’s lamp, showing the fluorescent emission of urine or amniotic fluid, could provide useful information regarding the disease etiology. The identification of porphyrin crystals in erythroid cells from bone marrow aspirate by an expert cytologist deserves special mention [16,17].

The recent presentations (see Table 5, XIV to XVII) are marked by the evolution of diagnostic tools, using the ‘genetic first’ approach, in which WES analysis is further confirmed by functional testing. The prevalence of severe CEP with perinatal presentation may increase in the coming years by revealing unexpected cases.

*UROS* gene analysis showed an unusual homogeneity with a predominant pathogenic variant, named C73R, which is known to be associated with severe manifestations [2]. The substitution located in the promoter region [24] and the various missense variants have all been shown to be deleterious. Concerning the novel variant (E75G, case XVII), given the severity of the phenotype and its vicinity regarding C73R’s position, the deleterious effect is unquestionable. The C73R mutant protein has been shown to have thermodynamic instability, affecting protein folding and causing premature degradation, as well as 70% of the missense variations described in CEP patients [7,28].

The most recent cases in this series are remarkable, with better outcomes than expected, paving the way towards potential therapeutic measures. As performed in alpha-thalassemia [29], regular transfusions from the intrauterine period, aiming to reduce ineffective erythropoiesis and early access to HSCT, are the main objectives. Regular and systematic transfusions from birth to transplant should improve engraftment by suppressing endogenous erythropoiesis and reducing plasmatic porphyrin levels as much as possible.

Although new therapeutic avenues have emerged in recent years, HSCT remains the reference treatment for severe forms of CEP disease. Approximately twenty cases of HSCT have been reported in the literature to date, and the most common indications were: (1) severe neonatal presentation, (2) chronic hemolysis with transfusion-dependent anemia, and (3) particular genotype associated with known severe forms (ex. Homozygous C73R) [30,31]. HSCT in CEP remains challenging, with very few eligible patients living long enough to benefit from the procedure. We recently reported the outcome of six CEP patients from the French cohort treated with HSCT [6]. The long-term efficacy of HSCT in CEP appears to be favorable, with near-normal porphyrin metabolism and the complete correction of cutaneous and hematological features. However, this study highlighted several important points regarding the safety and efficiency of HSCT in the context of CEP disease. First, primary graft failures were reported in half of the patients, even after standard myeloablative regimen. The most recent technologies allowing for T cell depletion from grafting should improve the success of haploidentical HSCT. Furthermore, the splenomegaly that is frequently observed in CEP patients with severe forms of the disease (Table 2 and Table 3) may promote HSC trapping and lower the preconditioning efficiency. Thus, HSCT in CEP has to strike a difficult balance between the need for myeloablation and the risk of regimen-related toxicity, especially hepatic disease. The second important feature is the prevalence of liver dysfunction, as revealed in our study (Table 2 and Table 3), and observed in most children before HSCT [6]. Liver involvement is unusual in CEP as compared with erythropoietic protoporphyria and the physiopathology remains to be elucidated. Liver biopsy specimens analyzed before HSCT showed porphyrin deposition in hepatocytes and Kupffer cells, which could induce liver fibrosis via tissue damage from porphyrin-mediated oxidative stress [6]. The most severe evolution seen in one patient included acute liver failure and severe GVHD, requiring several lines of immunosuppressive therapy, with grade III/IV skin, liver, gut, and eye involvement. This child simultaneously developed severe thrombotic microangiopathy and died of multiorgan failure at 6 months post-HSCT, despite treatment with monoclonal anti-C5 antibody (eculizumab). No veno-occlusive disease was observed.

These difficulties underline the importance of early diagnosis in these extremely rare patients. Finally, a well-defined registry including all severe CEP encountered worldwide is necessary to help diagnosis and ameliorate the therapeutic issues in the disease.

## Figures and Tables

**Table 1:** Main characteristics of severe antenatal forms of CEP.

Antenatal Cases	Hydrops (Weeks of Gestation)	Anemia	Splenomegaly	Hepatomegaly	Malformations	Biological Medium for Porphyrin Analysis	*UROS* Genotype	Abortion (Weeks of Gestation)	Transfusion (IU)	Premature Delivery	Viability	References
Family/ (Cases)	Main Pathological Features					Diagnosis		Outcome				
II	20 w	yes	yes	yes	no	Bd, Ur, Fe at birth	C73R/C73R		×2	32 w	no	[11]
IV	19 w	yes	yes	yes	mild	Am Fl/Fe Ti	C73R/-70T > C	19 w			no	[12,13]
VIII	17 w	yes	yes	yes	mild	Am Fl/Fe Ti	C73R/?	24 w			no	[13]
XIV (1 and 2)	19 w	yes	yes	yes	severe	NA (foetopathology only)	C73R/C73R	22/19 w			no	[14]
XVI			yes	yes	severe	BM, Bd, Ur	C73R/C73 R			35 w	yes	-
XVII	12 w			yes	mild	Fetal ascites	C73R/E75G	26 w			no	-

NA, not available; IU, in utero; *UROS* genotype cf Table 4; (?) unknown allele (exhausted sample), Am Fl, amniotic fluid; Bd, blood; BM, bone marrow; Fe, feces; Fe Ti, fetal tissues; Ur, urine; w, weeks).

**Table 2:** Main characteristics of severe neonatal forms of CEP.

Neonatal Cases	Respiratory Distress	Hemodynamic Shock	Hemorrhage	Photosensitivity	Hepato-Splenomegaly	Hemolytic Anemia	Thrombopenia	Liver Dysfunction	Porphyrins Analysis	UROS Genotype	Delivery (Weeks of Gestation)	Fatal Issue (Months of Life)	Transfusion	Aga at HSCT (Months of Life)	References
Family/(Cases)	Main Pathological Features					Biological Findings			Diagnosis		Outcome				
V	yes	yes	yes	yes	yes	yes	yes	yes	Bd, Ur	C73R/C73R	37 w	1 Mo	yes		[15]
XI	yes	yes	yes	yes	yes	yes	yes	yes	Bd, Ur, Fe	C73R/S212P	37 w		yes	10/43 Mo	[6]
XIII (1 and 2)	yes	yes	yes	yes	yes	yes	yes	yes	Bd	C73R/?	37/35 w	<3 Mo			
XV	yes	yes	yes	yes	yes	yes	yes	yes	Bd, Ur, BM	C73R/C73R	39 w		yes	10 Mo	[16,17]

*UROS* genotype cf Table 4; Bd, blood; BM, bone marrow aspirate; Fe, feces; Ur, urine; Mo, months.

**Table 4:** *UROS* gene analysis (HGVS denomination).

	Family/ (Cases)	UROS Genotype (NM_000375.3)			
		Allele 1		Allele 2	
**Antenatal**	**II**	c.217T > C	p.(Cys73Arg)	c.217T > C	p.(Cys73Arg)
	**IV**	c.217T > C	p.(Cys73Arg)	c.-70C > T	p.(?)
	**VIII**	c.217T > C	p.(Cys73Arg)	unknown	unknown
	XIV (1 and 2) *	c.217T > C	p.(Cys73Arg)	c.217T > C	p.(Cys73Arg)
	XVI *	c.217T > C	p.(Cys73Arg)	c.217T > C	p.(Cys73Arg)
	XVII *	c.217T > C	p.(Cys73Arg)	c.224A > G	p.(Glu75Gly) °
**Neonatal**	**V**	c.217T > C	p.(Cys73Arg)	c.217T > C	p.(Cys73Arg)
	**XI**	c.217T > C	p.(Cys73Arg)	c.634T > C	p.(Ser212Pro)
	**XIII-2**	c.217T > C	p.(Cys73Arg)	c.217T > C	p.(Cys73Arg)
	XV *	c.217T > C	p.(Cys73Arg)	c.217T > C	p.(Cys73Arg)
**Post-natal**	**I**	c.683C > T	p.(Thr228Met)	c.683C > T	p.(Thr228Met)
	**III**	c.217T > C	p.(Cys73Arg)	c.560A > C	p.(Gln187Pro)
	**VI**	c.217T > C	p.(Cys73Arg)	c.217T > C	p.(Cys73Arg)
	**VII (1 and 2)**	c.205G > A	p.(Ala69Thr)	c.205G > A	p.(Ala69Thr)
	**IX**	c.217T > C	p.(Cys73Arg)	c.205G > A	p.(Ala69Thr)
	**X**	c.673G > A	p.(Gly225Ser)	c.10C > T	p.(Leu04Phe)
	**XII**	c.205G > A	p.(Ala69Thr)	c.244G > T	p.(Val82Phe) °°

* WES analysis; ° unpublished *UROS* gene variant: °° splicing defect p.(?). The frequent missense variant C73R or p.(Cys73Arg) is shown in bold.

**Table 5:** Biochemical diagnosis from porphyrin metabolism analyses.

			Porphyrin Accumulation (Fold Increase vs. Normal)					
	Family/ (Cases)	Time Point	Urine			Blood		UROS Assay (% of Normal)
			Total	URO	COPRO	Plasma	RBC	
Antenatal	II	at birth	-	×1513	×360	×400	×17	-
	IV	still born	(1)	-	-	-	-	-
	VIII	still born	(2)	-	-	×885	-	25%
	XIV 1 and 2	still born	NA	-	-	-	-	-
	XVI	neonate	×1165	×1815	×715	* ×82	* ×30	* 62%
	XVII	still born	(3)	-	-	-	-	5% (4)
Neonatal	V	neonate	-	×1200	×142	-	×13	<1%
	XI	neonate	×597	×831	×370	×45	×21	20%
	XIII-2	neonate	-	-	-	-	×25	-
	XV	neonate	×116	×175	×93	* ×137	* ×24	* 77%
Postanatal	I	16 mo	×195	×770	×68	-	×30	37%
	III	8 mo	×187	×273	×144	-	×35	12%
	VI	8 mo	×328	×668	×158	-	×28	-
	VII-1	18 mo	-	×256	×30	-	-	5%
	VII-2	12 mo	-	×42	×25	-	-	5%
	IX	6 mo	×109	×202	×63	×5	×9	<2%
	X	6 mo	×1157	×2630	×421	×235	×72	15%
	XII	8 mo	×325	×476	×195	×33	×14	8%

(1) discoloured amniotic fluid with UV fluorescence; (2) porphyrins in amniotic fluid ×228 (URO-I, COPRO-I); (3) porphyrins in ascites fluid 343 nmol/L (URO I:85% URO III: 15%); (4) amniocytes; * after blood transfusion.

## Data Availability

Data will be made available by the authors upon request.

## References

[1] To-Figueras J., Millet O., Herrero C., Kadish K.M., Smith K.M., Guilard R. (2013). Congenital erythropoietic porphyria. Handbook of Porphyrin Science.

[2] Di Pierro E., Brancaleoni V., Granata F. (2016). Advances in understanding the pathogenesis of congenital erythropoietic porphyria. Br. J. Haematol..

[3] Irwin A.L., Desnick R.J. (2019). Congenital erythropoietic porphyria: Recent advances. Mol. Genet. Metab..

[4] Phillips J.D., Steensma D.P., Pulsipher M.A., Spangrude G.J., Kushner J.P. (2007). Congenital erythropoietic porphyria due to a mutation in GATA1: The first trans-acting mutation causative for a human porphyria. Blood.

[5] Ricci A., Di Betto G., Bergamini E., Buzzetti E., Corradini E., Ventura P. (2022). Iron Metabolism in the Disorders of Heme Biosynthesis. Metabolites.

[6] Besnard C., Schmitt C., Galmiche-Rolland L., Debray D., Fabre M., Molina T., Gouya L., Ged C., Castelle M., Cavazzana M. (2020). Bone Marrow Transplantation in Congenital Erythropoietic Porphyria: Sustained Efficacy but Unexpected Liver Dysfunction. Biol. Blood. Marrow. Transplant..

[7] Fortian A., González E., Castaño D., Falcon-Perez J.M., Millet O. (2011). Intracellular rescue of the uroporphyrinogen III synthase activity in enzymes carrying the hotspot mutation C73R. J. Biol. Chem..

[8] Urquiza P., Laín A., Sanz-Parra A., Moreno J., Bernardo-Seisdedos G., Dubus P., González E., Gutiérrez-de-Juan V., García S., Eraña H. (2018). Repurposing ciclopirox as a pharmacological chaperone in a model of congenital erythropoietic porphyria. Sci. Transl. Med..

[9] Egan D.N., Yang Z., Phillips J., Abkowitz J.L. (2015). Inducing iron deficiency improves erythropoiesis and photosensitivity in congenital erythropoietic porphyria. Blood.

[10] Bragazzi Cunha J., Elenbaas J.S., Maitra D., Kuo N., Azuero-Dajud R., Ferguson A.C., Griffin M.S., Lentz S.I., Shavit J.A., Omary M.B. (2021). Acitretin mitigates uroporphyrin-induced bone defects in congenital erythropoietic porphyria models. Sci. Rep..

[11] Verstraeten L., Van Regemorter N., Pardou A., de Verneuil H., Da Silva V., Rodesch F., Vermeylen D., Donner C., Noël J.C., Nordmann Y. (1993). Biochemical diagnosis of a fatal case of Günther’s disease in a newborn with hydrops foetalis. Eur. J. Clin. Chem. Clin. Biochem..

[12] Daïkha-Dahmane F., Dommergues M., Narcy F., Gubler M.C., Dumez Y., Gauthier E., Nordmann Y., Nessmann C., Terrasse G., Muller F. (2001). Congenital erythropoietic porphyria: Prenatal diagnosis and autopsy findings in two sibling fetuses. Pediatr. Dev. Pathol..

[13] Pannier E., Viot G., Aubry M.C., Grange G., Tantau J., Fallet-Bianco C., Muller F., Cabrol D. (2003). Congenital erythropoietic porphyria (Günther’s disease): Two cases with very early prenatal manifestation and cystic hygroma. Prenat. Diagn..

[14] Sudrié-Arnaud B., Legendre M., Snanoudj S., Pelluard F., Bekri S., Tebani A. (2021). An Atypical Case of Congenital Erythropoietic Porphyria. Genes.

[15] De Verneuil H., Moreau-Gaudry F., Ged C., Bensidhoum M., Hombrados I., Tricoire J., Rolland M. (1995). Fatal case of congenital erythropoietic porphyria in a neonate with acute hemolysis and hepatic failure. Arch. Pediatr..

[16] Saultier P., Loosveld M., Arnoux I., Tosello B., Quessada J., Barlogis V. (2021). Bone marrow erythroid cell inclusions reveal congenital erythropoietic porphyria. Br. J. Haematol..

[17] Khalouaoui A., Piarroux J., Sorin G., Oger M., Nicaise C., Tosello B. (2023). Perinatal onset of severe congenital erythropoietic porphyria. Arch. Dis. Child. Fetal. Neonatal..

[18] Zix-Kieffer I., Langer B., Eyer D., Acar G., Racadot E., Schlaeder G., Oberlin F., Lutz P. (1996). Successful cord blood stem cell transplantation for congenital erythropoietic porphyria (Gunther’s disease). Bone Marrow. Transplant..

[19] Dupuis-Girod S., Akkari V., Ged C., Galambrun C., Kebaïli K., Deybach J.C., Claudy A., Geburher L., Philippe N., de Verneuil H. (2005). Successful match-unrelated donor bone marrow transplantation for congenital erythropoietic porphyria (Günther disease). Eur. J. Pediatr..

[20] Lebreuilly-Sohyer I., Morice A., Acher A., Dompmartin A., Clement C., de Verneuil H., Ged C., Leroy D., Verneuil L. (2010). Congenital erythropoietic porphyria treated by haematopoietic stem cell allograft. Ann. Dermatol. Venereol..

[21] Lagarde C., Hamel-Teillac D., De Prost Y., Blanche S., Thomas C., Fischer A., Nordmann Y., Ged C., De Verneuil H. (1998). Allogeneic bone marrow transplantation in congenital erythropoietic porphyria. Gunther’s disease. Ann. Dermatol. Venereol..

[22] Thomas C., Ged C., Nordmann Y., de Verneuil H., Pellier I., Fischer A., Blanche S. (1996). Correction of congenital erythropoietic porphyria by bone marrow transplantation. J. Pediatr..

[23] Lienhardt A., Aubard Y., Laroche C., Gilbert B., Bernard P., Massri K., Bouleisteix J. (1999). A rare cause of fetal ascites: A case report of Günther’s disease. Fetal. Diagn. Ther..

[24] Solis C., Aizencang G.I., Astrin K.H., Bishop D.F., Desnick R.J. (2001). Uroporphyrinogen III synthase erythroid promoter mutations in adjacent GATA1 and CP2 elements cause congenital erythropoietic porphyria. J. Clin. Investig..

[25] Yasuda M., Desnick R.J. (2019). Murine models of the human porphyrias: Contributions toward understanding disease pathogenesis and the development of new therapies. Mol. Genet. Metab..

[26] Harteveld C.L., Higgs D.R. (2010). α-thalassaemia. Orphanet. J. Rare Dis..

[27] Millot S., Delaby C., Moulouel B., Lefebvre T., Pilard N., Ducrot N., Ged C., Lettéron P., de Franceschi L., Deybach J.C. (2017). Hemolytic anemia repressed hepcidin level without hepatocyte iron overload: Lesson from Günther disease model. Haematologica.

[28] Fortian A., Castaño D., Gonzalez E., Laín A., Falcon-Perez J.M., Millet O. (2011). Structural, thermodynamic, and mechanistical studies in uroporphyrinogen III synthase: Molecular basis of congenital erythropoietic porphyria. Adv. Protein Chem. Struct. Biol..

[29] Hui P.W., Pang P., Tang M.H.Y. (2022). 20 years review of antenatal diagnosis of haemoglobin Bart’s disease and treatment with intrauterine transfusion. Prenat. Diagn..

[30] Shaw P.H., Mancini A.J., McConnell J.P., Brown D., Kletzel M. (2001). Treatment of congenital erythropoietic porphyria in children by allogeneic stem cell transplantation: A case report and review of the literature. Bone Marrow Transplant..

[31] Díaz-Calvillo P., Rodríguez-Pozo J.Á., Martínez-López A., Aguilera-Peiró P., Díaz-de-Heredia C., Arias-Santiago S., Tercedor-Sánchez J. (2023). Twelve-year follow-up of bone marrow transplantation in congenital erythropoietic porphyria: Lessons learned from a challenging case. Clin. Exp. Dermatol..

